# Health Aspects, Growth Performance, and Meat Quality of Rabbits Receiving Diets Supplemented with Lettuce Fertilized with Whey Protein Hydrolysate Substituting Nitrate

**DOI:** 10.3390/biom11060835

**Published:** 2021-06-03

**Authors:** Ali Osman, Tharwat A. Imbabi, Abdalla El-Hadary, Islam Ibrahim Sabeq, Shimaa N. Edris, Abdel-Rahaman Merwad, Ehab Azab, Adil A. Gobouri, Amaal Mohammadein, Mahmoud Sitohy

**Affiliations:** 1 Department of Biochemistry, Faculty of Agriculture, Zagazig University, Zagazig 44511, Egypt; aokhalil@zu.edu.eg; 2 Department of Animal Production, Faculty of Agriculture, Benha Univerisity, Benha 13736, Egypt; Tharwat.mohamed@fagr.bu.edu.eg; 3 Department of Biochemistry, Faculty of Agriculture, Benha University, Benha 13736, Egypt; elhadary.a@fagr.bu.edu.eg; 4 Department of Food Hygiene, Faculty of Veterinary Medicine, Benha University, Benha 13736, Egypt; Islam.sabek@fvtm.bu.edu.eg (I.I.S.); Shimaa.edrees@fvtm.bu.edu.eg (S.N.E.); 5 Department of Soil Science, Faculty of Agriculture, Zagazig University, Zagazig 44511, Egypt; amerwad@yahoo.com; 6 Department of Nutrition and Food Science, College of Science, Taif University, P.O. Box 11099, Taif 21944, Saudi Arabia; e.azab@tu.edu.sa; 7 Department of Chemistry, College of Science, Taif University, P.O. Box 11099, Taif 21944, Saudi Arabia; a.gobouri@tu.edu.sa; 8 Department of Biology, College of Science, Taif University, P.O. Box 11099, Taif 21944, Saudi Arabia; amohamed@tu.edu.sa

**Keywords:** lettuce, nitrogen fertilization, whey protein hydrolysates, rabbits, growth performance, meat quality

## Abstract

Lettuce (*Lactuca sativa*) was grown using a foliar spray with whey protein hydrolysate (WPH) as opposed to normal nitrate fertilization. Lettuce juice was prepared from lettuce cultivated without any fertilization, nitrate fertilization, or WPH. Sixty weaned, 4-week-old male V-line rabbits with an average 455 ± 6 g body weight were randomly divided into 4 groups (*n* = 15) and administered different lettuce juices. Rabbits administered WPH-fertilized lettuce showed significantly higher (*n* = 5, *p* < 0.05) body weight and carcass weight than those receiving nitrate-fertilized lettuce. Rabbits administered nitrate-fertilized lettuce were associated with significantly (*p* < 0.05) higher levels of liver enzyme activities (AST, ALT, and ALP), bilirubin (total, direct, and indirect), and kidney biomarkers (creatinine, urea, and uric acid). Rabbits administered WPH-fertilized lettuce avoided such increases and exhibited normal levels of serum proteins. Rabbits administered nitrate-fertilized lettuce manifested significantly (*p* < 0.05) lower RBCs and Hb levels than that of the other groups, while those receiving WPH-fertilized lettuce showed the highest levels. Liver and kidney sections of rabbits receiving WPH-fertilized lettuce witnessed the absence of the histopathological changes induced by feeding on nitrate-fertilized lettuce and produced higher quality meat. WPH-lettuce can substitute nitrate-fertilized lettuce in feeding rabbits for better performance and health aspects.

## 1. Introduction

Attention is increasingly paid to the accumulation of nitrate in vegetables over fears of its adverse effect on human health. After entering the human body, bacteria and certain enzymes present in the human digestive system may convert nitrate to nitrite, which reaches the blood system where it oxidizes Fe^2+^ into Fe^3+^. Moreover, nitrite can react with amines and amides, forming nitrosamine and nitrosamide, well-known cancer-causing compounds [1]. About 72–94% of the total nitrate N is supplied to humans with daily vegetable diets [2]. For these reasons, many countries set up nitrate concentration limits for vegetables. In 1997, the European Union officially established maximum limits for nitrate in some vegetables, such as spinach and lettuce [3]. In China, Shen et al. [4] proposed 700 mg nitrate-N kg^−1^ as a maximum limit for leafy and root vegetables. Consequently, severe environmental challenges are generated, imposing a priority for producing new, safe nitrogen sources. The most promising candidates are still protein hydrolysates. Protein hydrolysates (PHs) are gaining prominence as bio-stimulants due to their potential to improve yield and nutritional quality, even under suboptimal nutrient regimens [5].

In the meat industry, nitrite is used as a curing agent to improve the flavor, taste, and aroma, preserve the meat’s red-pink color, and prevent the risk of bacterial contamination of cured meat, mainly from Clostridium botulinum [6]. Thus, most of the relevant publications illustrated the impact of nitrite on processed meat quality and the associated human health reflections. Conversely, the biological functions of protein hydrolysates, such as antimicrobial, antioxidant, antihypertensive, and immunomodulatory activities, and their nutritional value, oriented researchers to investigate them in animal nutrition [7].

The increasing application of nitrate fertilizers and curing agents raised concerns about their potential adverse effects on humans and the environment. However, the knowledge of nutrition’s health aspects on vegetables and fruits containing nitrate residues in mammalian systems is still limited. The present research investigated the health aspects, growth performance, and meat quality of rabbits reared on lettuce fertilized with whey protein hydrolysate (WPH) replacing nitrate. The comparison between different groups was conducted to explore differences in biochemical, hematological, and histopathological parameters, and meat quality of the treated animals.

## 2. Materials and Methods

### 2.1. Field Experiment

Field experiment was carried out on lettuce (*Lactuca sativa*) for the 2019 growing season in Hehia City, Sharkia Governorat, Egypt to study the effect of fertilization treatments with NH_4_NO_3_ and foliar spray with WHP (hydrolysed with pepsin at 37 °C for 8 h; degree of hydrolysis, 48%) on plant growth, yield, and nitrate content of lettuce and soil. Soil properties (as illustrated in Table 1) were analyzed according to Schuffelen et al. [8], Dinauer [9], and Jackson [10].

The experiment was designed in a randomized complete block in four replicates. Transplanting was carried out on 13 January 2019 on both sides of the ridges. The plot dimensions were 1.1 m^2^ width and 3.4 long and contained two rows 80 cm apart (3.4 m^2^ plot area; each row contained 24 plants 20–25 cm apart). Nitrogen treatment was added as ammonium nitrate NH_4_NO_3_ (33.5 %N) at a rate of 200 kg N ha^−1^ in 3 equal splits; the first was before the 1st irrigation (15 days after planting), while the second and third splits were 30 and 45 days after the first, respectively. Foliar spraying of WPHs was done in three equal doses at 15, 30, and 45 days after planting at a rate of 2 g L^−1^ (1.2 kg WPH/600 L water/ha). All plots received P and K. P was in the form of ordinary superphosphate, 65 g P kg^−1^, and added at 15 mg P kg^−1^ soil, and K was in the form of potassium sulfate added at 40 mg K kg^−1^ soil. P and K were added during soil preparation. Samples of plants were taken after 55 days to record vegetative plant characters, and fresh and dry weight. At harvest, five random plants per plot were taken, plant height and fresh weight were recorded, and the plants were then dried at 70 °C for 72 h. Assessment of NO_3_ in the lettuce was performed using Brucine method reported by Holty and Potworoski [11]. NO_3_ in soil was determined using the method described by Dinauer [9]. The current study data were subjected to an analysis of variance for a split block design after testing for the homogeneity of error variances. Statistically significant differences between means were compared at P ≤ 0.05 using Duncan’s multiple range tests. The statistical analysis was carried out using COSTAT computer software (CoHort Software version 6.303, Berkeley, CA, USA).

### 2.2. Animal Experiment

The current experimental procedures were carried out at the Rabbit Research Unit, Faculty of Agriculture, Benha University, Egypt. Animals were raised in compliance with the standards for husbandry derived from the standard operating procedures of the University of Benha. A total of 60 V-line weaned rabbits aged four weeks with a similar body weight average (450 ± 20 g) were divided into four groups (*n* = 15) and housed in twenty replicate cages (45 × 55 × 30 cm), with each holding three animals; five cages were randomly assigned to one of four treatments (as illustrated in Table 2). All animals were fed the same standard isocaloric/isonitrogenic diet during the experimental period (2 months). The basal diet composition and calculated analysis followed the nutrient requirements of rabbits from the National Research Council (as illustrated in Table 3).

#### 2.2.1. Growth Performance and Carcass Evaluation

The initial and final body weights (BW) were weekly recorded on an individual basis using a digital balance. The average daily weight gain (ADWG) was calculated as the difference between final and initial BWs divided by the number of days of the experimental period. At the end of the experimental period, a total of five animals (*n* = 5, the average weight of their group) were selected for all further experiments; the animals were slaughtered to evaluate the carcass traits and weight of internal organs. The weights of each carcass, its hind legs, saddle, thoracic neck, liver, kidney, spleen, and lung were recorded and expressed vis-à-vis the final body weight.

#### 2.2.2. Biochemical Blood Parameter Estimation

At the end of the experiment, 5 rabbits from each treatment were randomly taken after being fasted for 12 h and slaughtered for blood samples, carcass traits evaluation, and meat analysis. Blood samples were taken immediately after the slaughter of rabbits; two blood samples from each animal were taken into test tubes, the first containing EDTA, and the second without anticoagulant. Blood samples in the first tubes containing EDTA were used to perform complete blood pictures. The sample in the second tube (without anticoagulant) was transferred to a centrifuge tube and allowed to clot, centrifuged at 3000 rpm for 15 min (Centurion Scientific Ltd., K2015R, Church Farm, Stoughton, Chichester PO18 9JL, UK) to separate clear serum, which was stored at −20 °C until analysis. The separated serum was used for estimation of alanine amino transaminase (ALT), aspartate amino transaminase (AST), alkaline phosphatase (ALP), liver bilirubin (total, direct and indirect), total protein and serum albumin levels, and urea, uric acid, and creatinine levels according to the kit’s protocol of the manufacturer (Biodiagnostic Co., Giza, Egypt). Using an automatic cell counter (Cell-DYN 3500 Hematology Analyzer, Abbott Diagnostic Division, Santa Clara, CA, USA), the following components were determined in the first tube: the number of red blood corpuscles (RBC), hemoglobin concentration (Hb), hematocrit (Ht), the average volume of red blood corpuscles in the blood (MCV) and the total number of leukocytes (WBC), mean corpuscular hemoglobin (MCH), mean corpuscular hemoglobin concentration (MCHC), and platelets number. The results of the automatic cell counter were used to compare them with that of the results obtained by a manual method, where the results were largely the same [12].

Globulin was calculated as follows:

Globulin = total protein−albumin

#### 2.2.3. Histopathological Examination

The formalin-preserved rabbits’ liver and kidney specimens were processed in an automatic Microtome LAM-A10, Labtron Equipment Ltd., Camberley, UK. The processing consisted of two initial steps: fixation and dehydration. The organ was immersed in 10% buffered formalin for 48 h, followed by removal of fixative in distilled water for 30 min. Dehydration was carried out by successively running the tissues in a graded series of alcohol (70%, 90%, and 100%) followed by passing in several xylene changes. It consisted of tissue immersion for an hour in a mixture comprising 50% alcohol and 50% xylene, followed by pure xylene for one and a half hours. Samples were then impregnated with molten paraffin wax, embedded, and blocked out. Paraffin sections (4–5 μm) were stained with hematoxylin and eosin. Stained sections were examined for circulatory disturbances, inflammation, degenerations, apoptosis, necrosis, and any other pathological changes in the examined tissues.

#### 2.2.4. Meat Quality Estimation

Assessment of physicochemical attributes was conducted on rabbit meat samples from musculus Longissimus thoracis et lumborum (LTL), as outlined earlier [13,14]. The pH-meter (Jenway 3510 pH-meter, Cole–Parmer, Staffordshire, UK) was triply calibrated at three different pH points and then directly inserted into the LTL muscle to record the ultimate pH. The water holding capacity was measured by adding 5 kg to the preweighted portion of the rabbit LTL. The difference between the weight of intact meat and the pressed meat is expressed as the water holding capacity. LTL cuts of almost the same size (45 ± 2 gm) and shapes were used for the assessment of drip loss (48 h) percentages, where these portions were suspended over a plastic net in an airtight plastic box, preserved at 5 °C for 48 h, and then reweighted. Drip loss is measured as the amount of weight loss relative to the meat’s starting weight [15].

Similarly, the cooking loss percentages were also calculated, as revealed earlier by Honikel [15], where the targeted LTL cuts were separately loaded into thin-walled plastic thermo-tolerant bags and heated in a water bath until the monitored LTL core temperature reached 75 °C, then cooled to 5 °C in crushed ice, and reweighed to ascertain the cooking loss. Besides, cores were removed from cooked breast fillet samples for the Warner–Bratzler Shear Force (WBSF) evaluation using 3343 Universal Test System Mono column (Instron, USA) by cutting them perpendicular to the direction of the fiber [16]. One side of LTL was then incised longitudinally to estimate fresh meat color parameters, including lightness (L *), redness (a *), and yellowness (b *) by applying Chroma meter CR-410 (Konica Minolta Sensing INC., Osaka, Japan) 24 h postmortem. To estimate LTL color intensity chroma (c) and saturation (hue), the previously obtained values of redness (a *) and yellowness (b *) were applied to the following equations:

C=a∗^2+b∗^2^0.5)

ha=arctg b∗/a∗

An average of five measurements was calculated for each group. The evaluation test of the shelf-life was performed on the hind legs of the rabbit, as previously mentioned for chicken meat [14], with minor adjustment. Briefly, under aseptic condition, the bone was separated and the meat portions from the same group were mixed, and then 25 g of the meat mince was placed into a sterile falcon tube (50 mL). Three falcon tubes were specified for each checkpoint (5 checkpoints; day 1, day 3, day 5, day 7, and day 10) and then stored in a programmable incubator (Binder KB 23, BINDER GmbH (Headquarters), Tuttlingen, Germany) at 5 ± 0.2 °C through 10 days for further determination of the aerobic plate count (APC) and pH. APC was calculated in the same manner as previously demonstrated by Honikel [15], Silva et al. [16] and Sabike, Fujikawa [17] for natural beef microflora. This test’s pH was determined by the direct placing of the pH-meter glass electrode into the meat mince inside this falcon tube.

#### 2.2.5. Statistical Analysis

Shapiro–Wilk normality test was used to examine data distribution. The data were not normally distributed (*p* < 0.001 for Shapiro–Wilk test) except BW and ADWG. Hence, the nonparametric Kruskal–Wallis test was used to statistically analyze the differences among treatments. As the distribution of data was normal in case BW and ADWG, one-way statistical analysis was used. The data were presented as median, first, and third quartile (Q1, Q3) values in all data except for BW and ADWG, where the mean ± standard error (SE) was applied. All analyses were performed using SPSS (version 18.0).

## 3. Results

### 3.1. Field Experiment

Data presented in Table 4 show that plant height, fresh and dry weight, NO3- of lettuce plants were positively and significantly (*p* < 0.01) affected by applying NH_4_NO_3_ (200 kg N ha^−1^) and foliar spray with 2000 mg L^−1^ whey protein hydrolysates (WPH). The significantly (*p* < 0.05) highest values were observed at the ammonium nitrate treatment, while the lowest was obtained with control (without fertilization). On the other hand, the significantly (*p* < 0.01) highest values of accumulation NO_3_- in plants were observed with the application of ammonium nitrate (5858 mg kg^−1^ plants), against l (1343 mg kg^−1^ plants), in the unfertilized plants. Consequently, the content of nitrate in the ammonium nitrate-fertilized plants was about 4.4 times its content in the unfertilized plants. WPH fertilization kept the nitrate level in plants still low (2028 mg kg^−1^ plants), i.e., only about 1.5 times of the unfertilized plants. Even the unfertilized plants contain nitrate coming from the ordinary soil. This high level of plant nitrate content in response to nitrate fertilization may have health hazards, while the low level of nitrate in response to WPH fertilization may have protective health effect and refer to a safer substitute of nitrate fertilization. Generally, fertilization either with WPH or nitrate considerably and significantly (*p* < 0.01) enhanced the yield by about 32–38%.

### 3.2. Animals Experiment

#### 3.2.1. Growth Performance and Carcass Evaluation

The data in Table 5 were normally distributed as analyzed by Shapiro–Wilk. So, they were analyzed by one-way statistical analysis and expressed as the mean ± SE. As shown in Table 5, the results revealed that rabbits administered WPH-fertilized lettuce (group IV) showed increased BW8, BW12, ADWG 4–8, ADWG 8–12, and ADWG 4–12 (*n* = 5, *p* < 0.01, 0.05, 0.05, 0.05 and 0.05, respectively) compared to that of rabbits receiving nitrate-fertilized lettuce (group III) and other treatments, i.e., the negative and positive control (groups I and II). Rabbits’ administration with 10 mL of WPH-fertilized lettuce induced significant positive effects on the relative carcass weights of the cuts and internal organs (*n* = 5, *p* < 0.05), (as illustrated in Table 6). The group administered WPH-fertilized lettuce showed the highest (*n* = 5, *p* < 0.05) final live BW at 12 weeks, and also the highest carcass relative weight (*n* = 5, *p* < 0.05). It was considerably higher than the group administered nitrate-fertilized lettuce (Group III), representing 130% of its final live weight. Group III (receiving nitrate-fertilized lettuce) showed particularly and significantly lower carcass %, lower values of hind legs, thoracic, and fore legs weight and saddle (%) than that of the other groups weight (*n* = 5, *p* < 0.05, 0.05, 0.05 and 0.01, respectively). A significant (*p* < 0.05) reduction in the relative lung weight in group III (receiving nitrate-fertilized lettuce) against a significant increase in group IV (receiving WPH-fertilized lettuce) compared to that of the other control groups (*n* = 5, *p* > 0.05). Nonsignificant differences were observed in the relative weights of the other organs, i.e., kidney, heart, liver, and spleen (*n* = 5, *p* > 0.05).

#### 3.2.2. Biochemical Blood Parameter Estimation

##### Liver and Kidney Biomarkers

As shown in Table 7, rabbits administered nitrate-fertilized lettuce (group III) were associated with significant increases of liver enzyme (AST, ALT, and ALP) activities (*n* = 5, *p* < 0.01, 0.05, and 0.05, respectively), and smaller significant increases in bilirubin (total, direct, and indirect) than the other three groups (I, II, and IV) (*n* = 5, *p* < 0.05). In other words, rabbits administered WPH-fertilized lettuce were significantly lower than those receiving nitrate-fertilized lettuce in liver indicators but only slightly significantly lower the controls (groups I, II and III) in bilirubin total, direct, and indirect levels (mg/dL).

The data in Table 8 show that rabbits receiving 10 mL daily nitrate-fertilized lettuce (group III) were associated with significantly (*n* = 5, *p* < 0.05) higher levels of kidney biomarkers (creatinine and urea) than the group administered WPH-fertilized lettuce (group IV) (*n* = 5, *p*< 0.05), but they were not significantly different in the level of uric acid (*n* = 5, *p* > 0.05). On the other hand, group IV was significantly (*n* = 5, *p* < 0.05) lower than the other groups in (creatinine and urea), but not in uric acid (*n* = 5, *p* > 0.05). On the other hand, rabbits receiving nitrate-fertilized lettuce exhibited significantly lower levels of serum total protein, serum albumin, and serum globulin than that of the group administered WPH-fertilized lettuce (group IV) or the two controls (*n* = 5, *p* < 0.05, 0.01, and 0.05, respectively).

##### Hematological Parameters

Hematological parameters of rabbits administered lettuce juice fertilized with nitrate (group III) or whey protein hydrolysate (group IV) as compared to that of nonfertilized lettuce (group II) or of lettuce -free diet (group I) are presented in Table 9. Rabbits administered nitrate-fertilized lettuce (group III) manifested significantly lower levels of RBCs, Hb, HCT, PLT, and WBCs (*n* = 5, *p* < 0.001, 0.001, 0.05, 0.01, and 0.05) than that of the other groups (I, II, and IV). The group receiving WPH-fertilized lettuce (group IV) showed the highest levels of these two parameters (*n* = 5, *p* < 0.001, 0.001, 0.05, 0.01, and 0.05). MCV, MCH, MCHC, and RDW were not significantly (*n* = 5, *p* > 0.05) changed in all treatments

#### 3.2.3. Histopathological Findings

**Figure 1:**
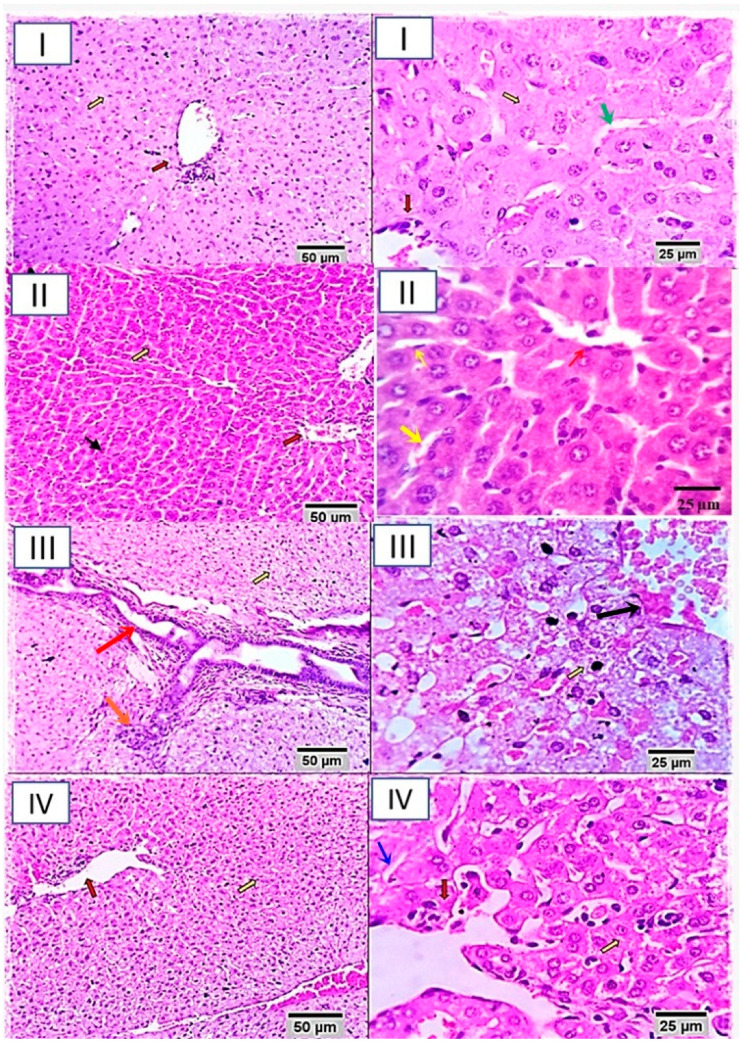
Photomicrograph from liver of different rabbit groups (I, II, III and IV) at 25 µm and 50 µm scale bars. Group I (healthy control, not receiving any supplement) shows preserved lobular arrangement, hepatic cord orientations (yellow arrows), portal triad structural components (red arrows), sinusoids, Von–Kuffer cells (green arrows), and stroma. Group II shows normal hepatic parenchyma with preserved hepatic cord arrangement (yellow arrows), portal triad structures (red arrows), and sinusoids (black arrow). Group III (rabbits receiving nitrate-fertilized lettuce) shows moderate portal and interstitial aggregations of round cells, mostly lymphocytes and plasma cells (orange arrow). Bile ducts appear moderately hyperplastic and appear to be suffering from chronic obstructive cholangitis (red arrow). Moderate portal vascular congestion and sinusoidal dilatation are seen (black arrow). Periportal hepatocellular degenerative changes (mostly hydropic degeneration) and early necrotic changes are seen (yellow arrows). Group IV shows degenerative changes in a few hepatocytes. Most of the degenerative changes were within the cloudy swelling and hydropic types (yellow arrows). Portal triads showed mild aggregation of lymphocytes and plasma cells (red arrows). Focally dilated hepatic sinusoids are seen (blue arrow).

**Figure 2:**
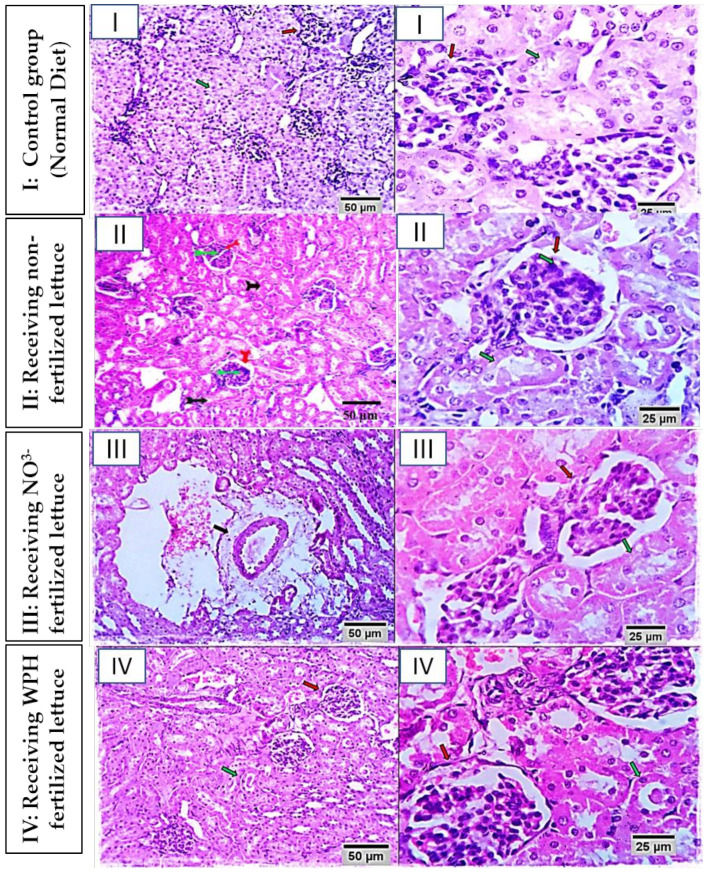
Photomicrographs from kidney of different rabbit groups (I, II, III, and IV) at 25 µm and 50 µm scale bars. Kidney sections of group I show normal cortical and medullary counterparts. Glomeruli, proximal, distal, and collecting tubules are normal (red and green arrows). Group II kidney tissues show normal nephron structural unites with preserved glomerular and tubular counterparts (red and green arrows). Group III kidney sections show moderate congestion of renal blood vessels and capillaries, marked perivascular edema, hemorrhages (black and blue arrows), and lobulated and partially atrophied glomeruli (red arrow). Some tubular epithelia appear degenerated, mostly cloudy swelling and hydropic degeneration (green arrow). Scale bars 50 µm and 25 µm for the images of liver and kidney sections, respectively. Group IV is showing lesions represented by mild congestion of the renal blood vessels (yellow arrow). Some renal tubules show mild cystic dilatation with atrophied epithelial lining, and others show intratubular hyaline casts (green arrows). A few glomeruli appear lobulated (black arrow). Scale bars 50 µm and 25 µm for images of liver and kidney sections, respectively.

Examined serial sections from liver and kidney of group I (rabbits fed on the basal diet without any treatments) revealed normal histomorphological structures of the examined organs. Liver sections showed preserved lobular arrangement, hepatic cords orientations, portal tirades structural components, sinusoids, Von–Kupffer cells, and stroma (as illustrated in Figure 1). Liver sections of group II (rabbits administered unfertilized lettuce juice) revealed normal hepatic parenchyma with preserved hepatic cords arrangement, portal triad structures, sinusoids, Von–Kupffer cells, and stroma. Liver sections of group III (rabbits fed on the basal diet + 10 mL lettuce juice (5.08 mg NO_3_), equivalent to 11.16 mg/kg bw/day, orally) revealed characteristic histopathological changes represented by moderate portal and interstitial aggregations of round cells, mostly lymphocytes and plasma cells. The bile ducts appeared moderately hyperplastic and suffered chronic obstructive cholangitis. Moderate portal vascular congestion and sinusoidal dilatation were seen. Periportal hepatocellular degenerative changes (mostly focally dilated hepatic sinusoids are observable in few sections). Figure 2 presents histopathological sections of the kidney of rabbits from the 4 studied groups (I, II, III, and IV). Kidney sections of group I exhibited apparently normal cortical and medullary counterparts. The glomeruli, proximal, distal, and collecting tubules were healthy. The renal papillae, pelvis, and stroma were normal. Kidney tissues of group II showed normal nephron structural unites with preserved glomerular and tubular counterparts. Kidney sections of group III showed moderate congestion of renal blood vessels and capillaries, marked perivascular edema and hemorrhages, lobulated and partially atrophied glomeruli, and focally, cystically dilated collecting and cortical tubules, sometimes with hyaline casts formation. Some of the tubular epithelia were degenerated, mostly cloudy swelling and hydropic degeneration. Kidney lesions in group IV samples were represented by mild congestion of the renal blood vessels; some of the renal tubules showed mild cystic dilatation with atrophied epithelial hydropic degeneration), and early necrotic changes were observed. Changes in group IV (rabbits administered juice of WPH-fertilized lettuce) were little. Liver sections manifested degenerative changes only in few hepatocytes. Most of the degenerative changes belong to the cloudy swelling and hydropic types. The portal tirades showed mild aggregation of lymphocytes and plasma cell lining, while others showed intratubular hyaline casts. A few glomeruli were lobulated.

#### 3.2.4. Meat Quality

Dietary lettuce juice treatments administered as unfortified lettuce or fortified with nitrate or protein hydrolysate (groups II, III, and IV) compared to that of the lettuce-free diet (group I) did not have significant impacts on the quality characteristics of the meat (as illustrated in Table 10), except for the Warner–Bratzler Shear Force (WBSF), the yellowness (b *), and the meat color saturation (hue) (*n* = 5, *p* < 0.01, 0.001, and 0.05, respectively).

On the other hand, the shelf-life test showed substantial variations in the aerobic plate count (APC) and pH value of stored rabbit meat based on supplementing the above-described lettuce treatments (as illustrated in Table 11). The meat from the groups receiving nitrate- or WPH-lettuce were significantly lower in APC than that of the controls after one-day and five-day storage (*n* = 5, *p* < 0.05), and significantly lower (*n* = 5, *p* < 0.01) after 3–10 days storages. The meat pH from the treated rabbit groups was not significantly different among the different groups at storage start and end (*n* = 5, *p* > 0.05), but it was significantly lower in the treated groups than that of the controls at storage days 3, 7, and 10 (*n* = 5, *p* < 0.01).

## 4. Discussion

Despite the high cost of nitrate and the potentially adverse environmental and health effects of nitrate leached into groundwater, it is still the most used mineral nutrient in horticulture industry [6]. To properly meet food demands and simultaneously enhance greenhouse systems’ sustainability, it is necessary to reduce the excessive inputs of nitrate fertilizers and improve nitrogen use efficiency while maintaining reasonable yield [7]. The use of natural plant biostimulants, including protein hydrolysates, was revealed to regulate and enhance crop resources use efficiency (RUE) and their ability to increase yields and abiotic stress tolerance [18,19]. The urgent need to reduce the use of nitrate fertilizers while increasing the NUE and maximizing crop productivity is a great challenge for modern agriculture. To participate in this trend, *L. sativa* was grown in the 2019 season to study the potential of using the foliar spray with WPH as a safer substitute to nitrate fertilization, while still maintaining good plant growth and yield and minimizing nitrate content in the edible leafy portion. The statistical analysis showed that foliar spray with 2000 mg L^−1^ WPHs significantly decreased the NO_3_- accumulated in plant and soil compared to that of the application of ammonium nitrate; these decreases represent 65% and 50%, respectively. This finding stands in agreement with those of Meier–Ploeger [20], who determined a lower nitrate concentration in cabbage with organic fertilization compared to that of mineral fertilization. Shahein et al. [21] detected a lower nitrate level in organically produced vegetables, including lettuce. Williams [22] reported lower nitrate concentrations in organically fertilized crops.

Nitrate levels in water and food supplies were so highly increased during recent decades worldwide that nitrate pollution became a global concern, affecting the food quality for daily use and impairing human and animal health [23,24,25]. Changes in liver enzymes (ALT, AST, and ALP) and kidney function (urea, creatinine, and uric acid concentration) in response to feeding on nitrate-fertilized lettuce (group III) were observed referring to hepatocyte necrosis, as confirmed by histopathology. The increased serum levels of hepatic enzymes could be attributed to the toxic effect of nitroso compounds formed in the stomach’s acidic environment, causing severe hepatic necrosis [26]. Rabbits administered nitrate (group III) also exhibited a significant decrease in TP, albumin, and bilirubin serum levels, probably because of the stimulation of thyroid and adrenal glands by sodium nitrate, leading to the blockade of protein synthesis, fast breakdown, increased rate of free amino acids, and decreased protein turnover [27]. Besides, the inhibition of protein synthesis also occurs because of nitric oxide release during nitrate toxicosis [28]. Alternatively, feeding rabbits on WPH-fertilized lettuce could avoid the increases in liver enzyme activities (AST, ALT, and ALP) probably due the potential protective action of the hydrolysate on liver function [29,30] and their general antibacterial activity maintaining the health aspect of the animals in good status [31,32,33,34].

The negative effect of nitrate salt on kidney function was confirmed by the increase in blood plasma urea, creatinine, and uric acid, and further confirmed by histopathological examination. Sharma et al. [35,36] indicated that nitrate impairs liver metabolism and kidney function. Several studies reported that excessive nitrate intake is a cause of many problems, such as methaemoglobinaemia [37]. The nitrate oxidative action may weaken the hematopoietic process, which produces new white blood cells (WBC) [16], or damages WBCs, reducing their count [38,39], in agreement with Sharma [40].

Hemoglobin is a significant source of free radicals in red blood corpuscles. It is a reactive molecule capable of gaining and losing electrons, giving rise to reactive species with the formation of methemoglobin [41] and consequent cell oxidative stress. The results revealed that nitrate-rich lettuce administration to female rabbits caused a reduction in the hemoglobin concentration in the blood, probably due to increased activity of the endothelial heme oxygenase by nitric oxide, degrading heme to carbon monoxide and biliverdin [42]. This result agrees with Gluhcheva [43], who suggested that the administration of nitrate caused a reduction in the red blood corpuscles count and decreased the concentration of the hemoglobin. The group receiving WPH-fertilized lettuce (group IV) showed the highest levels of these two parameters and general blood hematological parameters in accordance with [44].

The overall technological characteristics, including WHC, changes to drip loss (24 and 48 h), and cooking loss, as well as the appearance characteristics, including the lightness, redness, and chromium of rabbit meat (Longissimus thoracis et lumborum; LTL) did not improve due to any dietary supplementation. Current findings were consistent with those seen in broiler meat, where no significant effects of whey protein concentrate supplementation were noticed on water-holding capacity and cooking loss percentage [18]. Dietary whey administration did not have major effects on measured pH values in the rabbit or chicken muscles [22,23].

The WBSF value needed to shear meat from rabbit fed nonfortified lettuce juice (group II) and lettuce fortified with protein hydrolysate (group IV) was substantially lower than that of the control group and the group receiving nitrate-fertilized lettuce (III). Nevertheless, chroma and hue’s meat appearance, derived from control rabbits and rabbits supplied with WPH-fertilized lettuce (group IV), were redder than the other rabbit groups. Earlier studies indicated that whey protein concentrate did not affect broiler meat’s appearance and tenderness [18]. Low chroma and hue of rabbit meat receiving nitrate-fertilized lettuce juice in the current study may explain the significantly low hemoglobin levels and erythrocytes in this population.

On the other hand, the rabbits supplied with nitrate-fertilized lettuce juice (group III) provided the best keeping quality grade, which displayed the lowest pH values for ten days of storage. Compared to that of unfavorable antioxidant markers and haematological parameters, together with methemoglobinemia developed from the same group, this is an unexpected quality-result. The scarcity of literature on dietary effects of nitrate on meat quality makes it difficult to explain this phenomenon. However, the preservative effects of sodium and potassium nitrite and nitrate when directly added to meat products, such as reducing oxidative rancidity and inhibiting the development of some anaerobic microorganisms, may partially explain it [45]. Further, the protective effects of dietary nitrate on gastric epithelial integrity and intestinal microbiota may be another contributing factor [46]. WPH may be a good substitute of nitrate since meats from rabbits administered with WPH-fortified lettuce juice (group IV) achieved the second-highest rating, with lower APC and pH values than that of the negative control and the rabbits served nonfortified lettuce juice. These changes may be associated with powerful bioactive antimicrobial peptides of whey hydrolysates, which possibly triggered in the rabbit intestine by either hydrolytic digestive enzymes, proteolytic microorganisms, and/or the action of plant or microbial proteases [47,48,49,50,51]. Applying bioactive proteins and peptides was shown to have antibacterial and antioxidant benefits [52,53,54,55], for example, by increasing dietary WPC in broiler, resulting in a reduction in malondialdehyde (MDA) values and an improvement in glutathione peroxidase, glutathione-S-transferase, catalase, and superoxide dismutase in meat [56]. High antioxidant properties of whey are also attributable to the high content of biologically active peptides, amino acids, and glucose transporters, as well as to the promoted activity of enzyme production [57]. Besides, whey protein’s lactose content was reported to stimulate the development and spread of probiotic lactic acid bacteria in the intestine of rabbits. The associated depression of cecum pH due to lactose fermentation by cecal microflora can decrease potentially pathogenic bacteria [18,23]. Moreover, the better-quality indices in meat from rabbits administered nonfertilized lettuce juice than that of the negative control (not receiving any lettuce) may emit from the strong phenolic antioxidants, capable of neutralizing free radicals and the effective free radical scavenging properties [8,9]. In conclusion, the rabbits supplied with hydrolysate-fortified lettuce juice increased the quality of the meat and its shelf-life without affecting the performance of the rabbit compared to that of the nitrate-fortified lettuce juice feeding.

## 5. Conclusions

Rabbits administered WPH-fertilized lettuce could maintain significantly increased body weight during the whole rearing period, overcoming the observed reduction induced by feeding on nitrate-fertilized lettuce. This was reflected by significantly higher relative carcass weights of the cuts and internal organs of the rabbits receiving WPH-fertilized lettuce. The increases in liver enzyme activities (AST, ALT, and ALP), bilirubin (total, direct, and indirect), and kidney biomarkers (creatinine, urea, and uric acid) observed in rabbits receiving nitrate-fertilized lettuce were avoided in rabbits fed on WPH-fertilized lettuce. It can also increase blood RBCs and Hb levels, which is reduced by feeding on nitrate-fertilized lettuce. The liver and kidney sections of rabbits administered WPH-fertilized lettuce could be positively ameliorated by feeding on WPH-fertilized lettuce as substituting nitrate-fertilized lettuce. Rabbits supplied with WPH-fortified lettuce juice can produce higher quality meat than the rabbit receiving nitrate-fertilized lettuce juice. Investing more research in using protein hydrolysate as an environmentally friendly fertilizer may lessen the dependence on nitrate salt and participate in the production of higher yield and safer food quality products.

## Figures and Tables

**Table 1:** Some physical and chemical properties of investigated soil.

Soil Characteristics	Values
Soil particles distribution	
Sand,%	40.5
Silt,%	35.2
Clay,%	24.3
Textural class	Clay Loam
Field capacity (FC),%	22.9
CaCO_3_, (g kg^−1^)	15.1
Organic matter, (g kg^−1^)	3.6
pH *	7.92
EC, (dSm^−1^) **	1.45
Available N, (mg kg^−1^ soil)	62.5
Available P, (mg kg^−1^ soil)	12.6
Available K, (mg kg^−1^ soil)	194

* Soil-water suspension: 1:2.5. ** Soil water extract: 1:5.

**Table 2:** Experimental design for rabbit administered standard basal diet plus lettuce juice prepared from nonfertilized, nitrate-fertilized, and whey proteins hydrolysate (WPH)-fertilized lettuce fortified basal diet as compared to that of negative control, i.e., uniquely receiving basal diet.

No.	Groups	Main Diet	Supplement	Diet Nitrate from Lettuce (mg/kg bw/day)
I	Negative control (healthy animals)	Basal diet	—	NA
II	Non-fertilized lettuce	Basal diet+	10 mL lettuce juice (1.16 mg NO_3_)	2.5
III	Nitrate-fertilized lettuce	Basal diet+	10 mL lettuce juice (5.08 mg NO_3_)	11.16
IV	WPH-fertilized lettuce	Basal diet+	10 mL lettuce juice (1.76 mg NO_3_)	3.86

Every rabbit in groups II–IV received daily 10 mL lettuce juice by oral gavage during whole experimental period.

**Table 3:** Composition and calculated analyses of basal diet (g/kg, as-fed basis).

Ingredients, g/kg	Content
Alfalfa hay	330
Yellow corn	110
Soybean meal	96.9
Barely grain	90
Wheat bran	300
Wheat straw	50
Di-calcium phosphate	12.5
L-Lysine HCl	1.8
DL-Methionine	2.3
Sodium chloride	5
Vitamin/mineral premix ^1^	1.5
Total	1000.0
Calculated analysis (g/ kg, as-fed)	
Dry matter (DM), g/kg	914.41
Digestible energy (MJ/kg)	10.37
Crude protein (CP), g/kg	181.79
Crude fiber (CF), g/kg	135.36
Neutral detergent fiber (NDF), g/kg	348.88
Acid detergent fiber (ADF), g/kg	178.15
Ether extract (EE), g/kg	30.23
Ash, g/kg	65.30
Ca, g/kg	10.74
Available Phosphorus, g/kg	5.94
Methionine, g/kg	4.34
Lysine, g/kg	9.08

^1^ Minerals and vitamins mixture supply/kg of diet: 15,000 IU/kg vit. A, 2500 IU/kg vit. D3, 16.66 mg/kg vit. E, 2.0 mg/kg vit. K, 1 mg/kg vit. B1, 4 mg/kg vit. B2, 1.66 mg/kg vit. B6, 0.0034 mg/kg vit. B12, 6.66 mg/kg pantothenic acid, 1.07 mg/kg biotin, 1.66 mg/kg folic acid, 400 mg/kg choline chloride.

**Table 4:** Effect of fertilization treatments with NH_4_NO_3_ (200 kg N ha^−1^) and foliar spray with 2000 mg L^−1^. WPH on lettuce content of N-NO_3_ (mg kg^−1^ plant), growth, and yield traits.

Fertilization Treatments	N-NO_3_ mg kg^−1^ Soil	Plant Height (cm)	Fresh Weight (g plant^−1^)	Dry Weight (g plant^−1^)	Fresh Yield (Mg ha^−1^)	N-NO_3_ mg kg^−1^ Plant
Control	27.47 ± 0.90	25.37 ± 0.49	473.6 ± 1.83	38.03 ± 0.52	31.42 ± 0.25	1343 ± 4.27
NH_4_NO_3_ *	61.53 ± 0.68	37.17 ± 0.61	962.3 ± 2.81	64.73 ± 0.76	43.55 ± 0.60	5858 ± 2.50
WPH **	30.13 ± 0.56	34.7 ± 0.38	877.6 ± 1.04	56.23 ± 0.44	41.57 ± 0.31	2028 ± 3.58
*p*-value	0.0104	0.0052	0.0021	0.0021	0.0021	0.0021

* 200 kg N ha^−1^. ** 2000 mg L^−1^. All data are expressed as mean ± SE.

**Table 5:** Growth performance of weaned V-line rabbits administered lettuce juice fertilized with nitrate vs WPH as compared to that of nonfertilized lettuce or lettuce-free diet.

Growth Parameters	Lettuce Juice Supplementation				*p*-Value	Sig.
	I	II	III	IV		
BW4 (g)	455.0 ± 2.9	456.7 ± 4.4	455.0 ± 2.9	458.3 ± 4.4	0.9183	NS
BW8 (g)	843.3 ± 8.8 ^b^	982.7 ± 8.2 ^a^	983.3 ± 8.8 ^a^	1046 ± 32.1 ^a^	0.0017	**
BW12 (g)	1253 ± 8.8 ^b^	1599 ± 74.6 ^a^	1543 ± 66.9 ^a^	1635 ± 41.9 ^a^	0.0046	**
ADG4–8 (g/d)	18.87 ± 0.4 ^a^	18.79 ± 0.4 ^a^	13.87 ± 0.3 ^b^	21.00 ± 1.3 ^a^	0.0035	**
ADG8–12 (g/d)	20.00 ± 2.1 ^a^	22.01 ± 2.6 ^a^	14.64 ± 0.4 ^b^	21.02 ± 0.4 ^a^	0.0461	*
ADG4–12 (g/d)	19.43 ± 1.2 ^a^	20.40 ± 1.3 ^a^	14.26 ± 0.1 ^b^	21.01 ± 0.8 ^a^	0.0043	**

Data are expressed as the mean ± SE. Different superscript letters (^a^ and ^b^) in the same row refer to significant differences. NS: non-significant. * (*p* < 0.05, significant). ** (*p* < 0.005, highly significant). All rat groups received standard basal diet without (group I) or with (groups II-IV) 10 mL lettuce juice supplement. Lettuce juice (10 mL) was prepared from lettuce cultivated without (group II) or with nitrate fertilization (group III), or WPH (group IV) equivalent to 2.5. 11.16 and 3.86 mg/Kg bw/day, respectively. BW4, BW8, and BW12 is body weight (g) after 4, 8, and 12 weeks, respectively; DG4–8: average daily gain from 4–8 weeks; ADG8–12: average daily gain from 8–12 weeks; ADG4–12: average daily gain from 4–12 weeks.

**Table 6:** Median (Med), first, and third quartile (Q1 and Q3) for relative weights of carcass cuts and internal organs of weaned V-line rabbits administered nitrate- or WPH-fertilized lettuce juice (10 mL/animal/day) as compared to that of nonfertilized lettuce or lettuce-free diet (groups I and II, respectively).

Parameters (g)	Lettuce Juice Supplementation												*p*-Value
	I			II			III			IV			
	Q1	Med	Q3	Q1	Med	Q3	Q1	Med	Q3	Q1	Med	Q3	
Carcass (%)	40.7	43.8	46.1	47.0	47.3	50.4	47.3	47.6	48.2	48.1	48.2	48.5	0.0296
Hind legs (%)	15.6	15.7	16.1	15.2	15.7	18.4	18.3	19.2	19.5	18.2	18.5	18.9	0.0398
Saddle (%)	8.7	9.1	9.3	9.2	10.1	10.7	11.9	12.3	13.4	9.6	10.3	10.9	0.0030
Fore legs (%)	6.1	6.4	6.9	7.0	7.2	8.1	7.6	8.1	9.1	7.3	7.8	7.8	0.0398
Thoracic neck (%)	6.0	6.4	6.4	5.9	6.8	8.0	8.1	8.4	9.4	6.3	6.6	7.8	0.0418
Liver index	3.0	3.2	3.3	3.2	3.5	4.5	4.2	4.3	4.4	3.5	3.6	3.7	0.0702
Kidney index	0.5	0.6	0.7	0.6	0.6	0.7	0.7	0.7	0.7	0.6	0.6	0.6	0.3541
Heart index	0.3	0.4	0.5	0.4	0.5	0.5	0.4	0.4	0.5	0.3	0.3	0.4	0.3692
Lung index	0.6	0.6	0.6	0.6	0.6	0.6	0.6	0.6	0.7	0.5	0.5	0.5	0.0381
Spleen index	0.1	0.1	0.1	0.1	0.1	0.1	0.0	0.1	0.1	0.1	0.1	0.1	0.5514

All rabbit groups received diets explained in Table 2, and lettuce treatments were as follows: I: negative control (healthy animals); II: nonfertilized Lettuce; III: nitrate-fertilized lettuce; IV: WPH-fertilized lettuce. Parameters rate and organ index were calculated as follows: parameters rate or organ index = (organ weight/living weight) × 100%.

**Table 7:** Med, first, and third quartile (Q1 and Q3) of liver biomarkers in sera of rabbits administered nitrate- or WPH-fertilized lettuce juice (10 mL/animal daily) as compared to that of nonfertilized lettuce or lettuce-free diet (groups II and I, respectively).

Parameters	Lettuce Juice Supplementation												*p*-Value
	I			II			III			IV			
	Q1	Med	Q3	Q1	Med	Q3	Q1	Med	Q3	Q1	Med	Q3	
AST (U/L)	20.0	20.0	25.0	23	24	28	45	50	65	16	19	20	0.0014
ALT (U/L)	30.0	35.0	38.0	30	32	36	68	70	75	30	30	34	0.0294
ALP (U/L)	82.2	86.2	87.8	80.3	84.1	85.2	120.7	124.28	130.2	80.1	81.2	83.1	0.0109
Total BL * (mg/dL)	0.9	1.0	1.1	0.82	0.97	1.05	1.88	2.03	2.44	0.83	0.9	1.02	0.0454
Direct BL (mg/dL)	0.2	0.3	0.3	0.19	0.25	0.27	0.68	0.7	0.78	0.19	0.2	0.26	0.0450
Indirect BL (mg/dL)	0.7	0.7	0.8	0.63	0.72	0.78	1.2	1.33	1.66	0.64	0.7	0.76	0.0454

* BL; bilirubin. All rabbit groups received standard basal diet without (group I) or with 10 mL lettuce juice supplement (groups II–IV). Lettuce juice (10 mL) was prepared from lettuce cultivated without (group II), nitrate fertilization (group III), or WPH (group IV) equivalent to 2.5, 11.16, and 3.86 mg/kg bw/day, respectively. ALT: alanine amino transaminase; AST: aspartate amino transaminase, ALP: alkaline phosphatase.

**Table 8:** Med, first, and third quartile (Q1 and Q3) of protein profile and kidney biomarkers in sera of rabbits administered nitrate- or WPH-fertilized lettuce juice (10 mL/animal daily) as compared to that of nonfertilized lettuce or lettuce-free diet (groups II and I, respectively).

Parameters	Lettuce Juice Supplementation												*p*-Value
	I			II			III			IV			
	Q1	Med	Q3	Q1	Med	Q3	Q1	Med	Q3	Q1	Med	Q3	
Total protein (g/dL)	6.9	7.3	8.0	6.92	7.66	8.2	4.92	5.03	5.11	7.85	8.2	8.31	0.0114
Albumin (g/dL)	3.7	3.9	4.1	3.72	3.95	4.25	2.03	2.23	2.48	4.01	4.26	4.3	0.0088
Globulin (g/dL)	3.2	3.4	3.9	3.2	3.71	3.95	2.63	2.8	2.89	3.84	3.94	4.01	0.0181
Creatinine (mg/dL)	0.8	1.0	1.3	0.75	0.88	0.92	0.73	0.78	0.84	2.66	3.56	4.02	0.0109
Urea (mg/dL)	25.1	35.8	40.2	30.11	35.12	43.12	50.88	64.46	71.46	22.57	28.18	30.77	0.0181
Uric acid (mg/dL)	3.5	4.4	5.5	3.31	4.42	6.5	8.92	9.93	10.22	3.22	4.62	4.93	0.0674

All rabbit groups received standard basal diet without (group I) or with (groups II-IV) 10 mL lettuce juice supplement. Lettuce juice (10 mL) was prepared from lettuce cultivated without (group II) or with nitrate fertilization (group III), or WPH (group IV) equivalent to 2.5, 11.16, and 3.86 mg/kg bw/day, respectively.

**Table 9:** Med, first, and third quartile (Q1 and Q3) of hematological parameters in rabbits administered nitrate- or WPH-fertilized lettuce juice (10 mL/animal daily) as compared to that of nonfertilized lettuce or lettuce-free diet (groups II and I, respectively).

Hematological Parameters	lettuce Juice Supplementation												*p*-Value
	I			II			III			IV			
	Q1	Med	Q3	Q1	Med	Q3	Q1	Med	Q3	Q1	Med	Q3	
RBcs (× 10^6^/uL)	4.7	4.7	4.8	5.21	5.32	5.44	3.88	4	4.19	5.64	5.8	5.92	<0.001
Hb (g/dL)	10.4	10.5	11.1	10.8	11.24	12.65	8.33	9	9.11	12.8	13.24	15.25	<0.001
HCT (%)	32.6	35.7	40.1	31.1	35.49	40.11	24.08	26.14	29.7	35.9	39.18	42.16	0.0296
MCV(fL)	69.8	74.1	85.1	59.69	66.54	73.71	61.85	65.25	70.88	63.65	67.41	71.11	0.2849
MCH(Pg)	21.6	22.5	23.5	20.73	21.13	23.25	21.46	21.48	22.77	22.07	23.47	25.76	0.2638
MCHC(g/dL)	27.6	29.1	32.2	31.54	31.67	34.74	30.3	34.59	34.85	32.67	36.17	36.88	0.0989
RDW (%)	13.5	14.2	18.7	11.6	12.5	14.8	17.9	18.9	19.6	11.3	13.5	16.6	0.0603
PLT (×10^3^/uL)	388.0	400.0	410.0	409	415	420	300	310	329	405	420	425	0.0093
WBCs (×10^3^/uL)	0.94	0.94	1.3	1.48	1.59	1.71	0.34	0.46	0.65	2.05	2.21	2.33	0.0109

All rabbit groups received diets explained in Table 2, and lettuce treatments were as follows: I: negative control (healthy animals); II: non-fertilized lettuce; III: nitrate-fertilized lettuce; IV: WPH-fertilized lettuce. RBC: red blood cell; Hb: hemoglobin; HCT: hematocrit; MCV: mean corpuscular volume; MCH: mean corpuscular hemoglobin; MCHC: mean corpuscular hemoglobin concentration; RDW: red cell distribution width; PLT: platelet counts, WBC: white blood cell.

**Table 10:** Meat quality characteristics of rabbits administered lettuce juice fertilized with nitrate or WHP (groups III and IV, respectively) as compared to that of nonfertilized lettuce or lettuce-free diet (groups I and II, respectively).

Parameters	Lettuce Juice Supplementation				*p*-Value
	I	II	III	IV	
pHu	5.73 ± 0.08	5.65 ± 0.06	5.61 ± 0.05	5.64 ± 0.03	0.278
WHC	74.19 ± 2.53	79.05 ± 13.16	76.37 ± 1.05	81.67 ± 7.53	0.677
Drip loss (48 h)	5.50 ± 0.60	5.51 ± 1.26	8.90 ± 4.69	4.19 ± 0.58	0.194
Cooking loss	13.31 ± 1.36	10.33 ± 2.86	14.72 ± 3.48	9.75 ± 4.06	0.241
WBSF	4.93 ± 0.47	3.30 ± 1.21	4.42 ± 0.59	3.87 ± 1.21	0.005
L *	58.85 ± 1.88	62.69 ± 4.33	60.16 ± 1.74	63.06 ± 1.08	0.097
a *	10.59 ± 1.59	10.44 ± 2.00	9.77 ± 0.27	9.58 ± 0.95	0.673
b *	9.97 ± 0.95	7.57 ± 0.54	7.16 ± 0.35	8.38 ± 0.52	0.0002
Chroma	14.56 ± 1.62	12.91 ± 1.91	12.12 ± 0.24	12.75 ± 0.59	0.101
Hue	43.42 ± 3.59	36.30 ± 3.34	36.24 ± 1.80	41.29 ± 4.11	0.021

All data are expressed as mean ± SE. All rabbit groups received diets explained in Table 2, and lettuce treatments were as follows: I: negative control (healthy animals); II: nonfertilized lettuce; III: nitrate-fertilized lettuce; IV: WPH-fertilized lettuce. WHC: water holding capacity; WBSF: Warner–Bratzler Shear Force; L *: lightness; a *: redness, and b *: yellowness.

**Table 11:** Storage shelf life of rabbit minced meat stored for 10 days at 5 ± 0.2 °C. Meat samples were taken from rabbits. Administered lettuce juice fertilized with nitrate or WPH (groups III and IV, respectively) as compared to that of nonfertilized lettuce or lettuce-free diet (groups I and II, respectively).

	Lettuce Juice Supplementation				*p*-Value
	I	II	III	IV	
Storage Time (day)	Aerobic Plate Count (Log CFU/ g Meat)				
1	5.79 ± 0.07	4.50 ± 0.29	3.92 ± 0.04	4.72 ± 0.10	0.0255
3	5.70 ± 0.003	4.48 ± 0.00	3.80 ± 0.09	4.75 ± 0.06	0.0024
5	5.72 ± 0.16	4.22 ± 0.25	3.40 ± 0.10	4.55 ± 0.22	0.0112
7	6.84 ± 0.07	4.93 ± 0.008	3.74 ± 0.007	5.80 ± 0.007	0.0042
10	7.69 ± 0.11	6.25 ± 0.03	4.47 ± 0.04	5.65 ± 0.24	0.0042
pH					
1	5.66 ± 0.02	5.60 ± 0.007	5.61 ± 0.00	5.62 ± 0.007	0.0059
3	6.00 ± 0.02	5.79 ± 0.02	5.73 ± 0.07	5.83 ± 0.03	0.0157
5	5.87 ± 0.03	5.76 ± 0.01	5.63 ± 0.01	5.71 ± 0.007	0.0005
7	5.97 ± 0.01	5.86 ± 0.007	5.74 ± 0.01	5.85 ± 0.007	0.0112
10	5.9 ± 0.01	6.04 ± 0.14	5.90 ± 0.09	6.02 ± 0.19	0.7687

All data are expressed as mean ± SE. All rabbit groups received diets explained in Table 2, and lettuce treatments were as follows: I: negative control (healthy animals); II: nonfertilized lettuce; III: nitrate-fertilized lettuce; IV: WPH-fertilized lettuce. Aerobic plate count expressed as log CFU/g meat.

## Data Availability

Data available upon request.
