# Correction: Osman et al. Health Aspects, Growth Performance, and Meat Quality of Rabbits Receiving Diets Supplemented with Lettuce Fertilized with Whey Protein Hydrolysate Substituting Nitrate. *Biomolecules* 2021, *11*, 835

**DOI:** 10.3390/biom16050686

**Published:** 2026-05-06

**Authors:** Ali Osman, Tharwat A. Imbabi, Abdalla El-Hadary, Islam Ibrahim Sabeq, Shimaa N. Edris, Abdel-Rahaman Merwad, Ehab Azab, Adil A. Gobouri, Amaal Mohammadein, Mahmoud Sitohy

**Affiliations:** 1 Department of Biochemistry, Faculty of Agriculture, Zagazig University, Zagazig 44511, Egypt; 2 Department of Animal Production, Faculty of Agriculture, Benha Univerisity, Benha 13736, Egypt; 3 Department of Biochemistry, Faculty of Agriculture, Benha University, Benha 13736, Egypt; elhadary.a@fagr.bu.edu.eg; 4 Department of Food Hygiene, Faculty of Veterinary Medicine, Benha University, Benha 13736, Egypt; 5 Department of Soil Science, Faculty of Agriculture, Zagazig University, Zagazig 44511, Egypt; 6 Department of Nutrition and Food Science, College of Science, Taif University, P.O. Box 11099, Taif 21944, Saudi Arabia; e.azab@tu.edu.sa; 7 Department of Chemistry, College of Science, Taif University, P.O. Box 11099, Taif 21944, Saudi Arabia; a.gobouri@tu.edu.sa; 8 Department of Biology, College of Science, Taif University, P.O. Box 11099, Taif 21944, Saudi Arabia

In the original publication [1], there was a mistake in Figure 1 as published. An image of group IV was inadvertently duplicated and included in group II at 25 µm in Figure 1. The corrected Figure 1 appears below. The authors state that the scientific conclusions are unaffected. This correction was approved by the Academic Editor. The original publication has also been updated. 

## Figures and Tables

**Figure 1:**
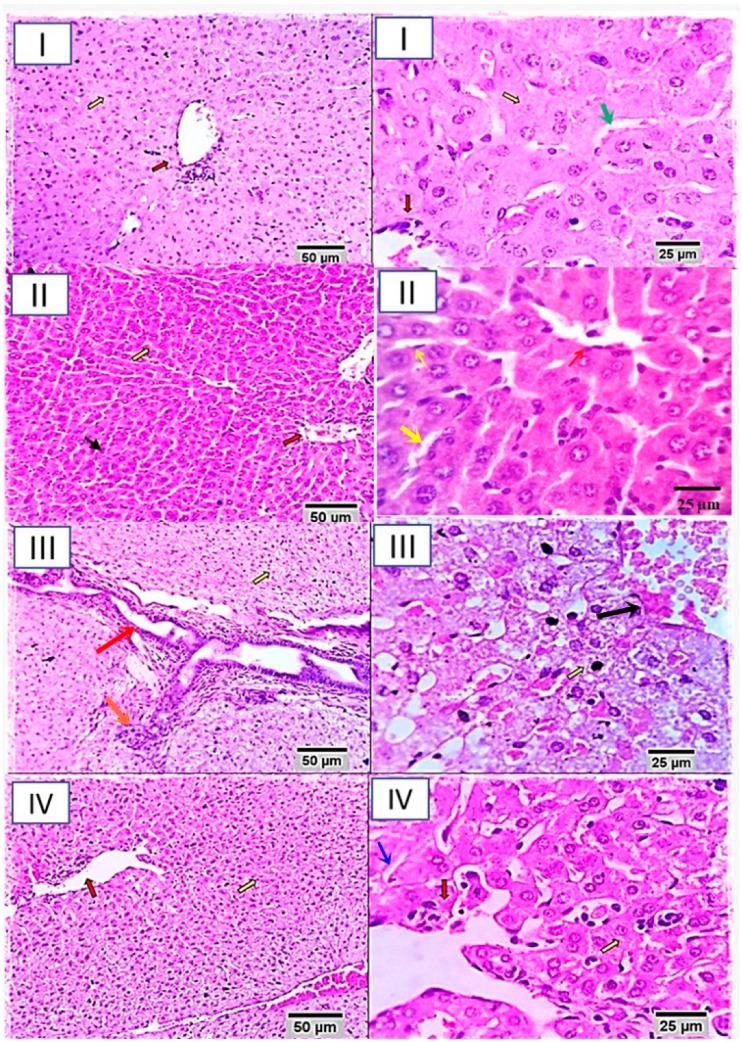
Photomicrograph from liver of different rabbit groups (I, II, III and IV) at 25 µm and 50 µm scale bars. Group I (healthy control, not receiving any supplement) shows preserved lobular arrangement, hepatic cord orientations (yellow arrows), portal triad structural components (red arrows), sinusoids, Von–Kuffer cells (green arrows), and stroma. Group II shows normal hepatic parenchyma with preserved hepatic cord arrangement (yellow arrows), portal triad structures (red arrows), and sinusoids (black arrow). Group III (rabbits receiving nitrate-fertilized lettuce) shows moderate portal and interstitial aggregations of round cells, mostly lymphocytes and plasma cells (orange arrow). Bile ducts appear moderately hyperplastic and appear to be suffering from chronic obstructive cholangitis (red arrow). Moderate portal vascular congestion and sinusoidal dilatation are seen (black arrow). Periportal hepatocellular degenerative changes (mostly hydropic degeneration) and early necrotic changes are seen (yellow arrows). Group IV shows degenerative changes in a few hepatocytes. Most of the degenerative changes were within the cloudy swelling and hydropic types (yellow arrows). Portal triads showed mild aggregation of lymphocytes and plasma cells (red arrows). Focally dilated hepatic sinusoids are seen (blue arrow).
